# Pregnancy outcomes and disease phenotype of hypertensive disorders of pregnancy in singleton pregnancies after in vitro fertilization: a retrospective analysis of 1130 cases

**DOI:** 10.1186/s12884-023-05838-5

**Published:** 2023-07-18

**Authors:** Fen Dai, Yehui Lan, Shuangjia Pan, Yuhuan Wang, Ying Hua, Wenya Xiao

**Affiliations:** Department of Obstetrics and Gynecology, The Second Affiliated Hospital of Wenzhou Medical University, Wenzhou, 325027 China

**Keywords:** In vitro fertilization, Hypertensive disorders of pregnancy, Severe preeclampsia, Early-onset preeclampsia

## Abstract

**Background:**

Although in vitro fertilization (IVF) can increase the incidence of hypertensive disorders of pregnancy (HDP), the pregnancy outcomes and disease phenotype of HDP in singleton pregnancies conceived via IVF remain unclear.

**Methods:**

This retrospective cohort study enrolled 1130 singleton pregnancies with HDP from 2016 to 2020. According to the mode of conception, they were allocated into IVF (n = 102) and natural conception (NC) groups (n = 1028). All IVF pregnancies were subdivided into frozen embryo transfer (FET) group (n = 42) and fresh embryo transfer (ET) group (n = 60). Demographic data, pregnancy outcomes and disease phenotypes of HDP among the groups were compared. The risk factors for severe preeclampsia (PE) and early-onset PE were analyzed.

**Results:**

The incidences of early-onset PE (*P<*0.001), severe PE (*P* = 0.016), cesarean section (*P<*0.001) and preterm births (*P =* 0.003) in the IVF-HDP group were significantly higher than those in the NC-HDP group, and gestational age at diagnosis of HDP (P = 0.027) and gestational age at delivery (P = 0.004) were earlier and birthweight of the neonates (P = 0.033) were lower in the IVF group. In singleton pregnancies with HDP, IVF was associated with increased risks for both severe PE and early-onset PE (aOR 1.945, 95% CI 1.256, 3.014; and aOR 2.373, 95% CI 1.537, 3.663, respectively), as well as FET, family history of preeclampsia, intrahepatic cholestasis of pregnancy, gestational hypothyroidism and multiparity were associated with increased risks of severe PE and early-onset PE.

**Conclusions:**

In singleton pregnancies with HDP, IVF was associated with an increased incidence of the disease phenotype (severe or early-onset PE), as well as an increased incidence of pregnancy outcomes related to severe PE and early-onset PE.

## Background

Hypertensive disorders of pregnancy (HDP), a group of common pregnancy complications, are categorized as follows: gestational hypertension, chronic hypertension, pre-eclampsia (PE), eclampsia, and PE super-imposed on chronic hypertension (superimposed PE) [1]. It complicates 3–10% of pregnancies globally [1] and accounts for 4.02-5.22% of all pregnancies in China [2]. The clinical presentation of HDP is various: from asymptomatic only hypertensive to immediate life-threatening complications including eclampsia, cerebral hemorrhage, placental abruption and a long-term cardiovascular disease postpartum [3–5]. It is one of the leading causes of pregnancy-related death worldwide [6] and low-income countries may suffer from a high burden of maternal death due to lack of access to adequate obstetric care [7]. It has been proposed that pregnancy outcomes of mild gestational hypertension are similar to those of the general obstetrics population [8, 9]. But preeclampsia (PE), presenting as new-onset hypertension with proteinuria or end organ dysfunction after 20 weeks’ gestation, or both, especially severe preeclampsia (PE with systolic blood pressure of 160 mmHg or diastolic blood pressure of 110 mmHg or more on two occasions at least 4 h apart) [1] or early-onset preeclampsia (< 34 weeks) had greater odds of maternal and fetal morbidities than mild gestational hypertension [10, 11]. The prognosis of HDP is closely related to the severity of disease process. Therefore, it is crucial to identify the disease phenotype of HDP.

At present, in spite of a massive research effort, the etiology of HDP is not completely understood. It has been acknowledged that the main pathophysiological feature of HDP is placenta dysfunction [12]. Similarly, in vitro fertilization (IVF), a widespread option for the treatment of human infertility, also was strongly associated with ischemic placental diseases [13]. Given similar placental pathomechanism, IVF has been considered to be a risk factor for HDP [2, 14]. There is accumulating evidence that singleton pregnancies who conceived via IVF had an increased risk of HDP [14, 15]. However, little attention has been paid to the effect of IVF on pregnancy outcomes and disease phenotype in pregnancies with HDP. A recent study analyzed the disease phenotype in preeclamptic singleton pregnancies after IVF, and found that IVF was associated with an increased risk for severe PE compared with those conceiving naturally [16]. Because of the small sample size and inadequate supports from other researches, it is still difficult to get a conclusion. In addition, it has been proposed that perinatal complications such as intrahepatic cholestasis of pregnancy (ICP) and gestational hypothyroidism are associated with an increased risk for severe PE [17, 18], which are the variables that should be taken into account when analyzing the effect of IVF on the disease phenotype of preeclampsia.

Hence, the objectives of this study were to investigate the differences in disease phenotypes (severe or early-onset PE) and pregnancy outcomes between HDP women after IVF and those after natural conception, and to further explore potential risk factors for severe or early-onset PE in HDP women after adjustment for confounders, including perinatal complications such as ICP and gestational hypothyroidism.

## Materials and methods

### Data sources and study population

This was a retrospective cohort study of all the singleton pregnancy women with HDP who were monitored prenatally from the first trimester and delivered at the Second Affiliated Hospital of Wenzhou Medical University between January 2016 and December 2020. Inclusion criteria were: singleton pregnancies with HDP and nonsmoking Han Chinese. We excluded the women with PE in a previous pregnancy and those complicated with diseases such as chronic hypertension, pregestational diabetes, pregestational thyroid, or kidney disease, autoimmune disease, as well as pregnancies conceived by ovulation induction, IVF with nonautologous oocyte-donation(OD) and intrauterine insemination. Participants were divided into the IVF group and the NC group according to the mode of conception. Those in IVF group were subdivided into fresh embryo transfer( ET) and frozen embryo transfer (FET) according to the type of transferred embryo, and were categorized into conventional IVF-ET and intracytoplasmic sperm injection (İCSİ) based on the different insemination methods. All the data were obtained from the electronic medical records by an experienced obstetrician, including the mode of conception, demographic features, IVF indications, embryo transfer type, insemination method, clinical indicators, obstetric complications, mode of delivery and neonatal information such as sex and birthweight of newborns, and the percentage of very low birthweight, neonatal asphyxia and admission to neonatal intensive care unit (NICU). The disease phenotypes of PE included mild and severe PE, early-onset PE, late-onset PE and eclampsia. Pregnancy outcomes included placental abruption, HELLP syndrome, gestational diabetes mellitus (GDM), gestational hypothyroidism, intrahepatic cholestasis of pregnancy (ICP), postpartum hemorrhage (PPH), intrauterine growth retardation, preterm birth and oligohydramnios.

### Relative definitions

The definition of HDP were based on the current criteria from ACOG (American College of Obstetrics and Gynecology) [1]. GDM was diagnosed by a 75 g oral glucose tolerance test [19]. The diagnose of ICP was established in presence of pruritus and elevated bile acids (> 10µmol/L) [20]. Gestational hypothyroidism was diagnosed when TSH levels > 2.5mIU/L in the first trimester or 3.0mIU/L in the second and third trimester [21]. Definition of other obstetric complications are as follows: PPH (blood loss more than 1000 ml in 24 h by Caesarean section or > 500 ml via vaginal delivery), neonatal asphyxia (5-minute Apgar score < 7), intrauterine growth restriction (IUGR, estimated fetal weight < 10th percentile or birthweight below the 10th percentile for gestational age derived from national growth curves), very low birthweight (birthweight < 1,500 g), placental abruption (premature separation of a normally implanted placenta before birth), oligohydramnios (four-quadrant amniotic fluid index < 5 cm or maximal deepest pocket < 2 cm), early preterm birth (< 34 completed gestational weeks) and late preterm birth (34^+ 0^−36^+ 6^ gestational weeks).

### Statistical analysis

Data analysis was performed using SPSS version 22.0 (SPSS, Statistical Package for the Social Sciences, IBM, NY, USA). We used Kolmogorov-Smirnov test to examine the normality of data and compared demographic data on mothers between IVF group and NC group by Parametric t-tests or non-parametric Mann-Whitney test, Chi-square test or Fisher exact test according to the feature of variables. We established a multiple logistic regressions model to evaluate severe PE and early-onset PE in relation to conception methods, as well as to identify potential risk factors. A *p-*value of less than 0.05 was considered as statistical significance.

## Results

**Fig. 1:**
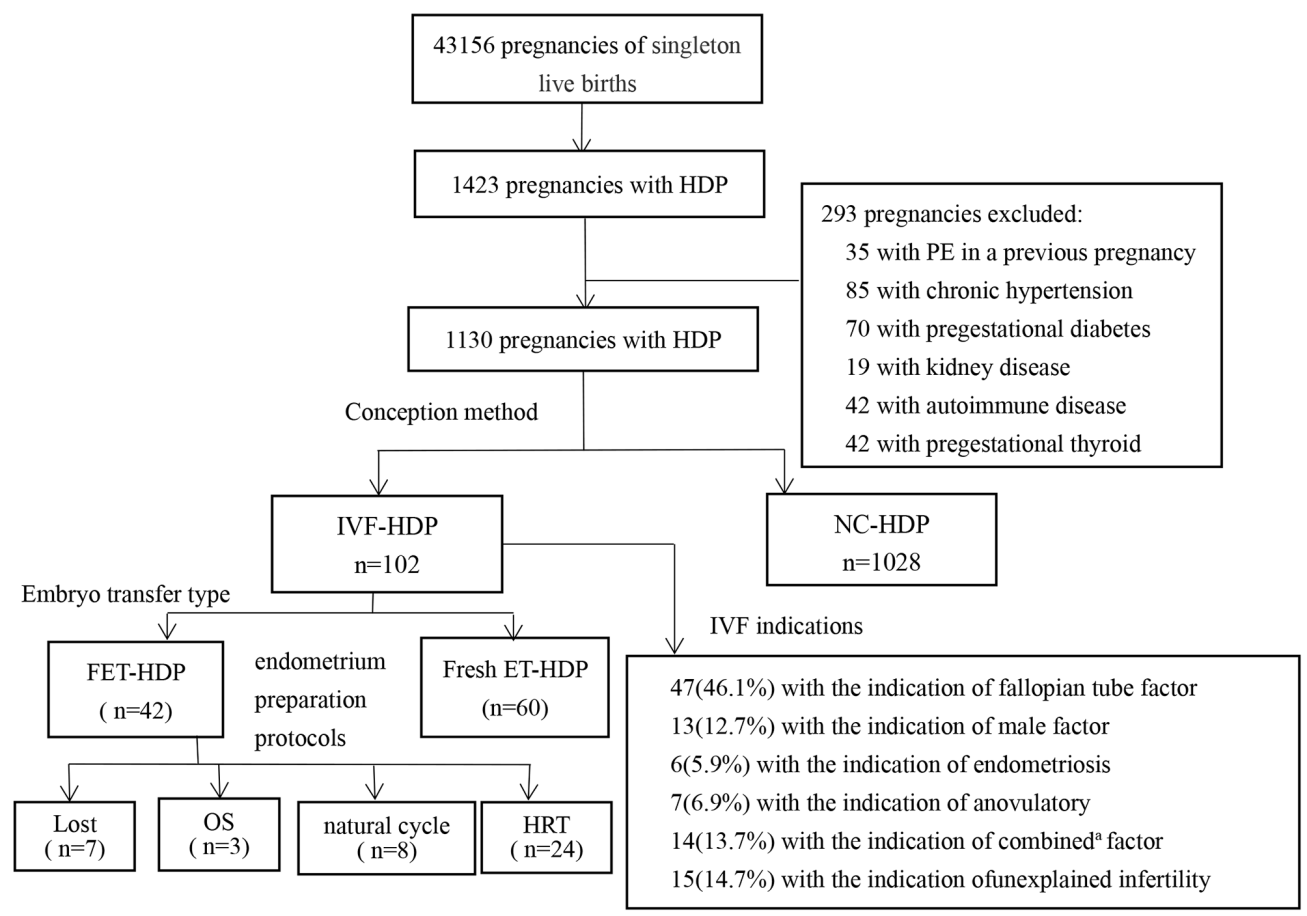
Flowchart of participants, IVF indications and the type of IVF in the study Abbreviations: HDP, hypertensive disorders in pregnancy; IVF, in vitro fertilization; NC, natural conception; ET, embryo transfer; FET, frozen embryo transfer; HRT,hormone replacement therapy; OS, ovarian stimulation; ^a^ combined was defined as two or more infertile causes mentioned Above

During the study period from 2016 to 2020, 41,156 singleton pregnancies gave birth in our hospital. Of these, 1660 ( 4.0%) pregnancies were complicated with HDP. According to the exclusion criteria, finally 1130 singleton pregnancies with HDP constituted the study group: 102 pregnancies via IVF and 1028 pregnancies via NC. Among 102 pregnancies with HDP in IVF group, fallopian tube factor, unexplained infertility and combined factors were the top three prevalent IVF indications. Of these, 60 (58.2%) women underwent fresh embryo transfer(ET) and 42 (41.2%) women with frozen embryo transfer (FET) based on the type of embryo transfer. All pregnancies with FET were categorized into three subgroups according to different endometrial preparation protocols, with 8 (19.1%) receiving natural cycles, 24 (57.1%) receiving hormone replacement therapy (HRT), 3 (7.1%) receiving ovarian stimulation (OS) protocols, and the rest (16.7%) missing information (Fig. 1). Of all pregnancies via IVF, only one woman was conceived by ICSI and the remaining conceived via conventional IVF-ET.

### Demographic data

The maternal baseline characteristics are showed in Table 1. Women in the IVF-HDP group were significantly older than those who conceived naturally [32.50 (30.00, 37.00) vs. 30.00 (26.00, 34.00); *P* < 0.001]. The rates of primipara and advanced maternal age in IVF-HDP group were higher than those in NC-HDP group (*P* < 0.05). There were no statistical differences between the two groups of women in terms of gravidity, pregnancy weight gain, the percentage of pregestational obesity, family history of preeclampsia and low-dose aspirin (LDA) usage (*P* > 0.05).

**Table 1:** Maternal characteristics of singleton pregnancies complicated with HDP via IVF or NC

	IVF-HDP group(n = 102)	NC-HDP group(n = 1028)	*P* value
Maternal age (years)	32.50(30.00, 37.00)	30.00(26.00, 34.00)	< 0.001
Advanced age(≥ 35 years) n (%)	37 (36.27)	243 (23.64)	0.005
Gravidity n (%)			0.546
1	38 (37.25)	374 (36.38)	
2	26 (25.49)	230 (22.37)	
3	20 (19.61)	180 (17.51)	
≥ 4	18 (17.65)	244 (23.74)	
Parity n (%)			< 0.001
0	81 (79.41)	542 (52.72)	
1	19 (18.63)	423 (41.15)	
≥ 2	2 (1.96)	63 (6.13)	
Pregestational obesity n (%)	8 (7.84)	90 (8.75)	0.755
Family history of preeclampsia n (%)	5 (4.90)	51 (4.96)	0.979
LDA usage n (%)	4 (3.92)	23 (2.24)	0.470

All data are expressed as the median [25–75 percentile] or n (%) ;

Abbreviations: IVF, in vitro fertilization; NC, natural conception; PE, preeclampsia; LDA, low-dose aspirin.

Disease phenotypes and perinatal outcomes were summarized in Table 2. Compared with women in the NC-HDP group, both gestational age at diagnosis (median 34.07 vs. 36.43 weeks, *P* = 0.027) and gestational age at delivery (median 37.43 vs. 38.29 weeks, *P* = 0.004) in the IVF-HDP group were much lower, as a result, arising the higher incidences of early-onset PE (49.02% vs. 31.03%, *P* < 0.001) and preterm births (45.10% vs. 30.54%, *P* = 0.003), especially preterm delivery at 34 to 34 + ^6^ weeks (10.78% vs. 5.16%, *P* = 0.019), and the lower birthweight (median 2690.00 vs. 2980.00 g, *P* = 0.033). Besides, the prevalence of severe PE (50.98% vs. 38.72%, *P* = 0.016) and cesarean Sect. (87.25% vs. 61.09%, *P* < 0.001) was significantly higher in the IVF-HDP group than those in the NC-HDP group. Differences in the rates of gestational hypertension and other obstetric complications such as GDM, gestational hypothyroidism, ICP, oligohydramnios, HELLP syndrome, placental abruption and eclampsia were small and unlikely to be clinically significant. There were also no significant differences in neonatal outcomes including the rate of sex, very low birthweight, neonatal asphyxia, and NICU admission between the two groups (*P* > 0.05).

**Table 2:** Disease phenotype and pregnancy outcomes of singleton pregnancies of IVF-HDP and NC-HDP group

	IVF-HDP group (n = 102)	NC-HDP group (n = 1028)	*P* value
Gestational age at delivery (weeks)	37.43(34.29, 39.00)	38.29(36.14, 39.43)	0.004
Gestational age at diagnosis (weeks)	34.07(30.68, 38.21)	36.43(32.18, 38.57)	0.027
Gestational hypertension n (%)	13 (12.75)	197 (19.16)	0.112
PE n (%)	89 (87.25)	831 (80.84)	0.112
Mild PE n (%)	37 (36.27)	433 (42.12)	0.253
Severe PE n (%)	52 (50.98)	398 (38.72)	0.016
Early-onset PE n (%)	50 (49.02)	319 (31.03)	< 0.001
Late-onset PE n (%)	39 (38.24)	512 (49.81)	0.026
Placental abruption n (%)	1 (0.98)	31 (3.02)	0.385
HELLP syndrome n (%)	1 (0.98)	31 (3.02)	0.385
Eclampsia n (%)	0 (0.00)	6 (0.58)	1.000^*^
Maternal complications			
PPH n (%)	7 (6.86)	65 (6.32)	0.831
Gestational diabetes mellitus n (%)	30 (29.41)	220 (21.40)	0.063
Gestational hypothyroidism n (%)	9 (8.82)	83 (8.07)	0.792
Intrahepatic cholestasis during pregnancy n (%)	7 (6.86)	46 (4.47)	1.000
Oligohydramnios n (%)	9 (8.82)	66 (6.42)	0.352
Intrauterine growth retardation n (%)	16 (15.69)	158 (15.37)	0.933
Preterm delivery n (%)	46 (45.10)	314 (30.54)	0.003
Preterm delivery (34–34 + 6weeks) n (%)	11 (10.78)	53 (5.16)	0.019
Preterm delivery (35–35 + 6weeks) n (%)	8 (7.84)	41 (3.99)	0.117
Preterm delivery (36–36 + 6weeks) n (%)	9 (8.82)	70 (6.81)	0.447
Preterm delivery (< 34weeks) n (%)	18 (17.65)	150 (14.59)	0.408
Cesarean section n (%)	89 (87.25)	628 (61.09)	< 0.001
Perinatal outcome			
Birthweight (g)	2690.00 (2077.50, 3297.00)	2980.00 (2320.00, 3400.00)	0.033
Male neonatal n (%)	57 (55.88)	520 (50.58)	0.307
Very low birthweight n (%)	21 (20.59)	145 (14.11)	0.078
Neonatal asphyxia n (%)	6 (5.88)	66 (6.42)	0.832
Admission to NICU n (%)	43 (42.16)	339 (32.98)	0.062

All data are presented as the median [25–75 percentile] or n (%) ;

Abbreviations: IVF, in vitro fertilization; NC, natural conception; PE, preeclampsia; HELLP syndrome, hemolysis, elevated liver enzymes, and low platelet count; NICU, neonatal intensive care unit; PPH, postpartum hemorrhage;

**Fig. 2:**
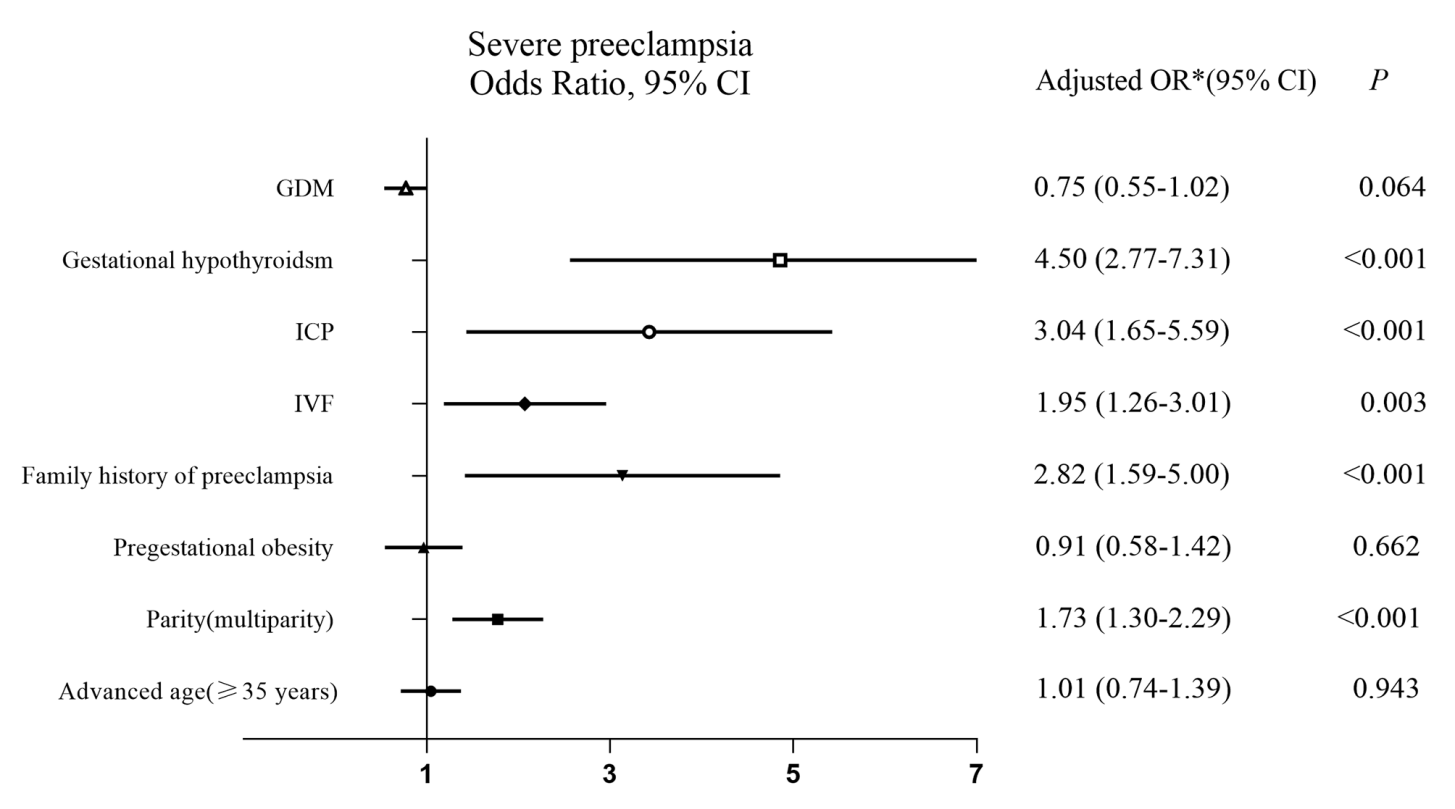
Forest plot of risk factors for severe preeclampsia in singleton pregnancy complicated with HDP Abbreviations:OR, odds ratio; aOR, adjusted odds ratio; CI,confidence interval; IVF,in vitro fertilization; ICP, intrahepatic cholestasis of pregnancy; GDM, gestational diabetes mellitus; ^*^Adjustments for maternal advanced age (≥ 35 years), parity (multiparity vs. primiparity), pregestationa obesity, family history of preeclampsia, ICP, IVF, gestational hypothyroidism and GDM

**Fig. 3:**
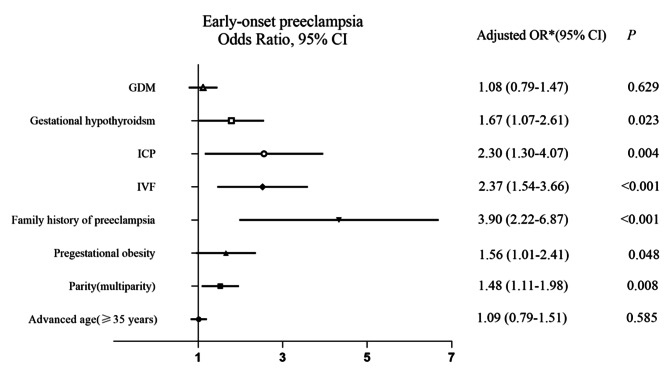
Forest plot of risk factors for early-onset preeclampsia in singleton pregnancy complicated with HDP Abbreviations:OR, odds ratio; aOR, adjusted odds ratio; CI,confidence interval; IVF,in vitro fertilization; ICP, intrahepatic cholestasis of pregnancy; GDM, gestational diabetes mellitus; ^*^Adjustments for maternal advanced age (≥ 35 years), parity (multiparity vs. primiparity), pregestationa obesity, family history of preeclampsia, ICP, IVF, gestational hypothyroidism and GDM

Multivariable logistic regression analysis was further performed to identify the risk factors for severe PE and early-onset PE, which was presented in Figs. 2 and 3. IVF was associated with an increased risk for both severe PE and early-onset PE (aOR 1.945, 95% CI 1.256, 3.014, *P* = 0.003; and aOR 2.373, 95% CI 1.537, 3.663, *P* < 0.001, respectively) after adjustment for confounding factors including advanced age, parity, pregestational obesity, family history of preeclampsia and perinatal complications such as gestational hypothyroidism, ICP and GDM. Family history of preeclampsia (aOR 2.821, 95% CI 1.594, 4.991, *P* < 0.001), ICP (aOR 3.041, 95% CI 1.654, 5.590, *P* < 0.000) and gestational hypothyroidism (aOR 4.496, 95% CI 2.766, 7.308, *P* < 0.001) were associated with an increased risk of severe PE, but advanced age and pregestational obesity were not. Likewise, family history of preeclampsia (aOR 3.904, 95% CI 2.217, 6.873, *P* < 0.001), ICP (aOR 2.300, 95% CI 1.302, 4.065, *P* = 0.004), gestational hypothyroidism (aOR 1.673, 95% CI 1.073, 2.606, *P* = 0.023) and pregestational obesity (aOR 1.555, 95% CI 1.005, 2.406, *P* = 0.048) were associated with an increased risk of early-onset PE, but advanced age was not (aOR 1.094, 95% CI 0.793, 1.510, *P* = 0.585). Interestingly, multiparity was associated with an increased risk of both severe PE (aOR 1.728, 95% CI 1.304, 2.291, *P* < 0.001) and early-onset PE (aOR 1.481, 95% CI 1.109, 1.980, *P* = 0.008).

In a subgroup analysis of the effect of embryo transfer type on disease phenotype, women with HDP in the FET group had higher risks of early-onset PE and severe PE (OR 4.455, 95% CI 1.508, 7.915; OR 3.589, 95% CI 1.556, 8.279, respectively) than those in the fresh-ET group. After adjustment for confounding factors including maternal advanced age (≥ 35 years), primiparity, pregestationa obesity, family history of preeclampsia, ICP, gestational hypothyroidism and GDM, the risk for both early-onset PE and severe PE remained (aOR: 4.341, 95% CI: 1.730-10.896, P = 0.002; aOR: 3.949, 95% CI: 1.571–9.926, P = 0.003, respectively) in FET group (Table 3).

**Table 3:** Effect of FET on the risks of early-onset PE and severe PE by logistic regression analysis

outcomes	Unadjusted OR(95% CI)	aOR^*^(95% CI)	*P* value
early-onset PE	3.455 (1.508–7.915)	4.341 (1.730-10.896)	0.002
severe PE	3.589 (1.556–8.279)	3.949 (1.571–9.926)	0.003

Abbreviations: PE, preeclampsia;OR, odds ratio; aOR, adjusted odds ratio; CI, confidence interval; FET, frozen embryo transfer;

^*^Adjustments for maternal advanced age (≥ 35 years), primiparity, pregestationa obesity, family history of preeclampsia, intrahepatic cholestasis of pregnancy, gestational hypothyroidism and gestational diabetes mellitus.

## Discussion

Our study found that pregnancies with HDP who conceived through IVF had a significantly increased risk for developing severe PE and early-onset PE, as well as increased pregnancy outcomes associated with severe PE and early-onset PE, including preterm delivery, lower birthweight and cesarean section, compared to those conceived naturally. Furthermore, co-existing risk factors for severe PE and early-onset PE included FET, family history of preeclampsia, ICP, gestational hypothyroidism and multiparity.

The underlying mechanism by which IVF is associated with severe PE or early-onset PE is unclear. Abnormal placentation might be one reason [3, 13]. Early-onset pre-eclampsia arises predominantly owing to defective placentation during the first few weeks of pregnancy because it is characterized by poor remodeling of the uteroplacental spiral arteries, and has been defined as a placental type PE. By contrast, late-onset preeclampsia appears to be driven by the interactions between normal senescence of the placenta and a maternal genetic predisposition to cardiovascular and metabolic disease, which has been defined as maternal type PE [12]. It has been proposed that IVFET process may alter gene and protein expression in placental tissues during the first trimester, thereby arising the risk of early-onset pre-eclampsia [22].

Another important evidence is the imbalance in the circulating concentrations of angiogenic factors such as soluble fms-like tyrosine kinase1 (sFLT-1) and placental growth factor (PlGF), leading to impaired trophoblast invasion, subsequent reduced vascular remodeling and placenta hypoperfusion [23]. Several studies have demonstrated that IVF singleton pregnancies are associated with an increased antiangiogenic profile (elevated sFlt-1 and decreased PlGF) at multiple time points throughout gestations when compared with those conceived spontaneously [24, 25]. It is believed that an increase in the ratio of sFLT-1:PlGF in circulating concentration was correlated with the severity of PE, and the more intense the imbalance in angiogenic factors, the greater the severity of the PE [26]. Besides, serum sFLT-1 level is significantly elevated in patients with preeclampsia, especially in those with early-onset preeclampsia [27].

In addition, Our study suggested that FET increased the risk of early-onset PE and severe PE, which was partly consistent with the previous findings [28]. Different protocols used for endometrium preparation in FET cycles including hormone replacement therapy (HRT) maybe contribute to the increased risk of severe PE in IVF-HDP pregnancies. The lack of the corpus luteum, which secretes vasoactive hormones such as relaxin, in HRT cycles may, at least in part, result in the observed increased risk of PE and PE with severe features [28, 29]. It is a pity that we cannot do this subanalysis according to the endometrial preparation protocols in the FET group because some data about endometrial preparation protocols were missing and the sample size was too small to explore associations between different endometrial preparation protocols and disease phenotypes of HDP.

It was reported that the risk of PE was elevated in women with infertility-related diagnoses such as tubal, ovulatory factor, polycystic ovary syndrome(PCOS) and endometriosis compared with those with natural conception [30]. Thus, the cause of infertility may play a role in the occurrence of the PE. For example, prior studies have suggested that endometriosis was associated with an increased risk of pre-eclampsia, as patients with endometriosis had a different endocrine activation of macrophages that mediate apoptosis of extravillous trophoblasts, a proposed pathway leading to preeclampsia development [31, 32]. In addition, the women with PCOS are commonly conceived via IVF, who are susceptible for severe pre-eclampsia because they are more susceptible to metabolic disorders and lower insulin sensitivity [33, 34]. Based on these findings, we assume that the mechanism of the effect of IVF on different phenotype of PE may be mediated by the coexistence of multiple factors and not by a single cause.

Family history of preeclampsia has been acknowledged as a risk factor for PE [35]. However, only a few scholars have focused on the effect of family history of preeclampsia on disease phenotype of PE [36, 37]. Their findings were consistent with ours that a maternal family history of preeclampsia was associated with an increased risk of not only early-onset PE, but also severe PE. This may be that large heritable component influencing the development of preeclampsia is present in families with clustering of hypertensive disorders [35, 38].

To our knowledge, this is the first study to investigate the relationship between early-onset PE and some perinatal complications such as ICP and gestational hypothyroidism. Our data showed that ICP and gestational hypothyroidism were associated with the increased incidence of early-onset PE and severe PE. The results of associations between severe PE and ICP or hypothyroidism were in line with some prior studies [39, 40]. Possible mechanisms are vasoconstriction induced by high bile acid levels or partial chronic endothelial cell injury due to abnormal thyroid hormone levels, all of which may account for the higher prevalence of severe PE [40, 41]. These results need to be supported by further research.

Interestingly, we found that multiparity appeared to be a risk factor of both severe and early-onset PE, while advanced maternal age and obesity were not. These contradictory results may be due to different study designs and study population. Our study focused on the patients who were diagnosed with HDP, while previous studies population was involved in normotensive pregnancies. Hence, our research proposed a hypothesis that in the HDP pregnancies, obesity, advanced age, parity may play different roles in disease phenotype of HDP.

The strength of our study is that we provide a new perspective to analyze the association between IVF and HDP by exploring the differences in disease phenotypes (severe or early-onset PE) and pregnancy outcomes between HDP women after IVF and those after natural conception, and further explore potential risk factors for severe or early-onset PE in HDP women. In addition, we took account of confounding variables such as ICP and gestational hypothyroidism, which had often been ignored in previous studies. However, as a single-center, retrospective analysis, this study has several limitations. First, some clinical data, such as income, social status, baseline sex hormoneand the stage of transferred embryo, etc., were not available from the cases. Second, the relationship between the ICSI and disease phenotypes of HDP was not available because only one woman in the IVF group underwent ICSI. Third, we did not list specific biochemical markers for assessing the severity of disease in both groups. Further prospective studies are needed to better identify the influence of IVF on the disease phenotype and pregnancy outcomes in women with HDP.

## Conclusions

In conclusion, our results suggested IVF implicated disease phenotypes and pregnancy outcomes of HDP, including severe or early-onset PE and some adverse outcomes such as higher rate of cesarean delivery and preterm birth, and lower birthweight of neonates. Furthermore, our study found that co-existing risk factors for severe PE and early-onset PE had family history of preeclampsia, ICP, gestational hypothyroidism and multiparity. Therefore, close follow-up and surveillance for preeclampsia in pregnant women via IVF is necessary, especially for those with these risk factors.

## Data Availability

The datasets used and/or analyzed during the current study are available from the corresponding author on reasonable request.

## Abbreviations

ACOG: American College of Obstetrics and Gynecology
ART: Assisted reproductive technology
BMI: Body mass index
CVD: Cardiovascular disease
CL: Corpus luteum
ET: Embryo transfer
FET: Frozen embryo transfer
GDM: Gestational diabetes mellitus
HDP: Hypertensive disorders of pregnancy
HELLP: Hemolysis, elevated liver enzymes, and low platelet
HRT: Hormone replacement therapy
ICP: Intrahepatic cholestasis of pregnancy
IVF: In vitro fertilization
LBW: Low birth weigh
LDA: Low-dose aspirin
NC: Natural conception
OS: Ovarian stimulation
PCOS: Polycystic ovary syndrome
PE: Preeclampsia
PlGF: Pacental growth factor
PPH: Postpartum hemorrhage
SFlt-1: Soluble fms-like tyrosine kinase-1
